# Parental Preferences about Policy Options Regarding Disclosure of Incidental Genetic Findings in Newborn Screening: Using Videos and the Internet to Educate and Obtain Input

**DOI:** 10.3390/ijns8040054

**Published:** 2022-09-27

**Authors:** Michael H. Farrell, Katherine E. Mooney, Anita Laxova, Philip M. Farrell

**Affiliations:** 1 Departments of Internal Medicine and Pediatrics, University of Minnesota Medical School, Minneapolis, MN 55455, USA; 2 Department of Medicine, University of Wisconsin School of Medicine and Public Health, Madison, WI 53792, USA; 3 Department of Pediatrics, University of Wisconsin School of Medicine and Public Health, Madison, WI 53792, USA; 4 Departments of Pediatrics and Population Health Sciences, University of Wisconsin School of Medicine and Public Health, CSC Room K4/948, 600 Highland Avenue, Madison, WI 53792, USA

**Keywords:** cystic fibrosis, incidental findings, CFTR-related metabolic syndrome, cystic fibrosis screen positive inconclusive diagnosis, newborn screening, next generation sequencing, policy

## Abstract

Our objective was to develop and test a new approach to obtaining parental policy guidance about disclosure of incidental findings of newborn screening for cystic fibrosis (CF), including heterozygote carrier status and the conditions known as CFTR-related metabolic syndrome (CRMS) and/or cystic fibrosis screen positive inconclusive diagnosis, CFSPID. The participants were parents of infants up to 6 months old recruited from maternity hospitals/clinics, parent education classes and stores selling baby products. Data were collected using an anonymous, one-time Internet-based survey. The survey introduced two scenarios using novel, animated videos. Parents were asked to rank three potential disclosure policies—Fully Informed, Parents Decide, and Withholding Information. Regarding disclosure of information about Mild X (analogous to CRMS/CFSPID), 57% of respondents ranked Parents Decide as their top choice, while another 41% ranked the Fully Informed policy first. Similarly, when considering disclosure of information about Disease X (CF) carrier status, 50% and 43% gave top rankings to the Fully Informed and Parents Decide policies, respectively. Less than 8% ranked the Withholding Information policy first in either scenario. Data from value comparisons suggested that parents believed knowing everything was very important even if they became distressed. Likewise, parents preferred autonomy even if they became distressed. However, when there might not be enough time to learn everything, parents showed a slight preference for deferring decision-making. Because most parents strongly preferred the policies of full disclosure or making the decision, rather than the withholding option for NBS results, these results can inform disclosure policies in NBS programs, especially as next-generation sequencing increases incidental findings.

## 1. Introduction

### 1.1. Cystic Fibrosis Newborn Screening

Cystic fibrosis (CF) newborn screening (NBS) has been performed in the United States for over 30 years [1], and in some European regions such as Veneto, Italy for almost 50 years [2]. The protocols have changed over time, especially during the past decade with nationwide programs underway [3,4]. The original protocols used a first tier of immunoreactive trypsinogen (IRT) analysis followed by a second IRT [5], and later a DNA-based test was introduced for the p.Phe508del (F508del) variant of the cystic fibrosis transmembrane conductance regulator (*CFTR*) gene [6]. Subsequently, the DNA tier was expanded to between 23 and 372 pathogenic variants [7,8,9]. When a single *CFTR* variant is detected in the DNA tier, sweat testing is essential because in up to 10% of such cases a second, undetected *CFTR* variant may be present to cause the disease [6]. NBS also produces incidental findings (IFs), namely detection of more babies who are heterozygote carriers [8,9,10], and others have a condition known as CFTR-related metabolic syndrome (CRMS) or CF screen positive, inconclusive diagnosis (CFSPID) [11,12]. Many IFs lack significance for the child’s health and may lead to misconceptions, emotional complications, and biomedical risks because of unnecessary tests or treatments [13,14]. NBS policy has been crafted by public health experts and sometimes informed by bioethics commentary, surveys of parents, and advocacy by laboratory methodologists [1,15,16,17]. Many variations in CF NBS algorithms have resulted from these efforts [3], rarely with input by parents.

### 1.2. Next Generation Sequencing in Newborn Screening

Next-generation sequencing (NGS) technologies [8,9] are now available and will help to increase NBS sensitivity, i.e., the percentage of CF cases identified. However, NGS also produces more IFs. Thus, the application of NGS may lead to more psychosocial complications. NBS programs are looking for ways to mitigate harm as they increase the benefits through NGS.

Thus, the pivotal introduction of NGS with its unprecedented technology has reinvigorated the longstanding debate about whether NBS programs should notify parents about IFs, given that the risk/benefit ratio is uncertain [14,18]. CF carrier status and CRMS/CFSPID are unlike most NBS results as they do not require immediate medical attention, although these conditions are often disclosed with counseling in the neonatal period. However, some programs do not ensure that IFs are disclosed. In fact, at least one country (Norway) by law does not reveal *CFTR* carrier status discovered through NBS [4]. In the USA, many IF results are returned to the primary care provider who may lack sufficient time, knowledge or counseling skill [19,20], and may not even know the family because of inaccurate or insufficient labeling of dried blood spot specimens [21,22]. Therefore, parents can become anxious or confused about the implications of the results, as has been noted after NBS and other community screening programs [23,24]. Infants with CRMS/CFSPID may also have had biomedical complications of tests or treatments, which might have been unnecessary [11,12]. Since NGS and the increased number of IFs may cause a change in the balance of risks and benefits of NBS, it is important to re-examine policies and responsibilities for reporting results.

### 1.3. Policy Options for Disclosure

After reviewing the limited literature on this topic, we decided that it would be important to obtain fresh perspectives from new parents about potential policies. We were aware of three potential policy options for communicating IFs, namely Fully Informed, Withhold Information, and Parents Decide (see descriptions in Table 1). We sought to develop a survey instrument to gather parents’ policy advice about two research questions: (1) how should NBS programs communicate with parents about single-variant NBS results that are consistent with being a carrier?; (2) how should NBS programs communicate with parents about one or two mutations consistent with a mild version of the screened disease, which has minor health significance compared to the full disease (e.g., CRMS/CFSPID)? Our hypotheses were based on three decades of NBS follow-up experience and especially our recent studies [21,23,24,25,26], suggesting parents would wish to know about IFs even if the information was complex and potentially stressful and even if the condition was mild.

## 2. Materials and Methods

### 2.1. Design

The study used an anonymous online survey that contained three animated video clips, each of which explained some background information necessary for understanding the questions. The survey was hosted by Qualtrics (Provo, Utah and Seattle, Washington, DC, USA). Participants could complete the survey using a computer, smartphone, or iPad with Internet access. IRB approval was obtained from Aurora Health Care in Milwaukee, Wisconsin, the University of Wisconsin School of Medicine and Public Health, and Meriter Hospital in Madison Wisconsin. Parents were recruited to take the survey predominantly in Madison, after an initial effort had limited success in Milwaukee. Consent was obtained online from each participant before they began the survey.

### 2.2. Methodologic Elements to Support the Objectives

To increase the utility of the study for policy making, we included several innovations in the design. These resulted from sequential quality improvement efforts to create a user-friendly, unbiased survey of parental opinions during the first six postpartum months.

#### 2.2.1. Embedded Explanatory Videos

**Figure 1:**
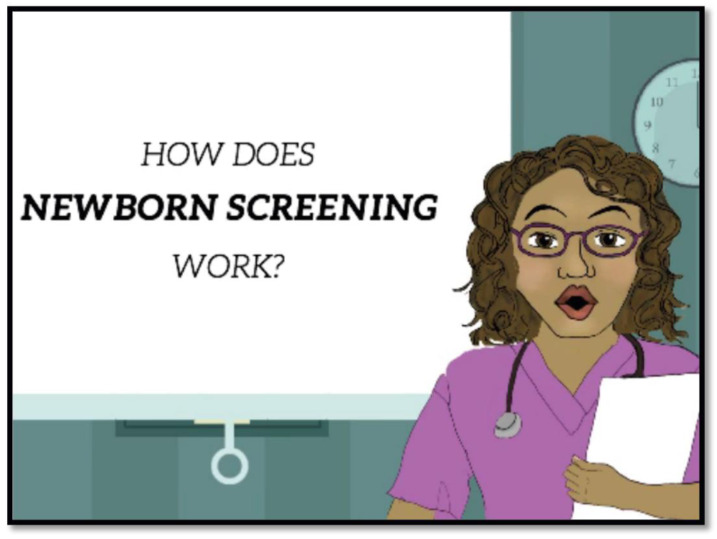
Still Image from the introductory video featuring “Nurse Maria” and explaining. the process and benefits of newborn screening Full video available at https://www.youtube.com/watch?v=2qeBX0FDp_I&t=3s [last accessed on 25 July 2022].

**Figure 2:**
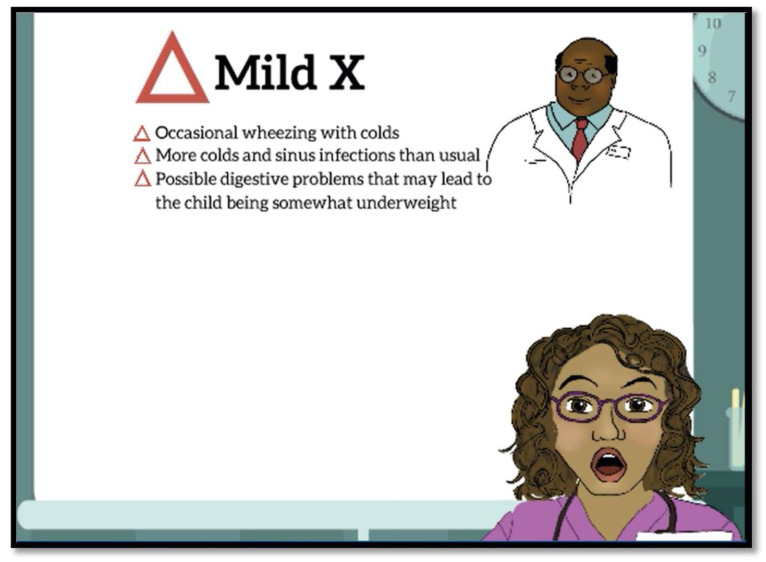
Still Image from the second video depicting symptoms of “Mild X” (analogous to CRMS/CFSPID). Full video available at https://www.youtube.com/watch?v=3HiFPywnCb0 [last accessed on 25 July 2022].

**Figure 3:**
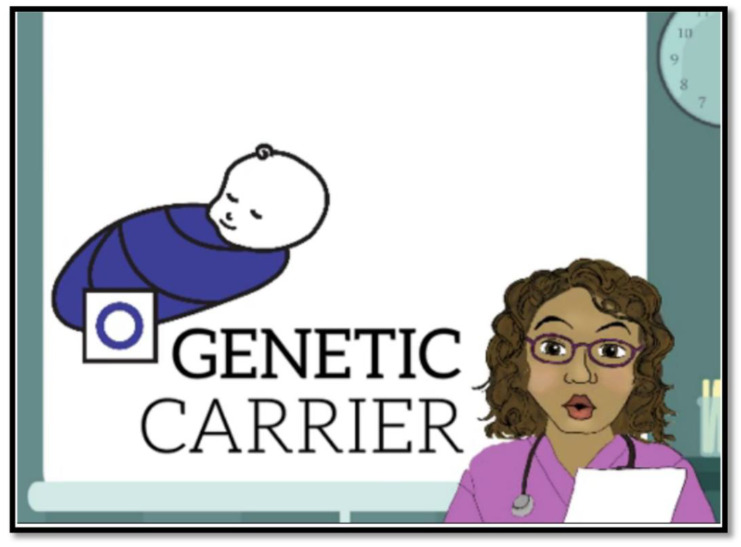
Still image from the third video detailing the meaning of carrier status. for “Disease X”. Full video available at https://www.youtube.com/watch?v=Y572EiX_hWY [last accessed on 25 July 2022].

Preference and opinion surveys often present several sentences of background information to read before asking questions. During our survey instrument’s development, we became concerned about the amount of text that would be needed before asking key questions. We therefore created three animated video clips embedded between sections of the survey (Figure 1, Figure 2 and Figure 3). The videos featured an animated character, Nurse Maria, who explained the basics of NBS and presented different scenarios for disclosure of NBS results. The videos were scripted in stages to support a careful order of survey questions, as described below. Each video lasted about 5 min. The language was assessed and determined to be appropriate for those with an eighth-grade education.

The video’s script and graphics were drafted and vetted with a variety of parents and NBS educators so that they would be accessible to participants regardless of prior education and medical experience. The animations were revised and pilot tested before routine use in this study. All videos were uploaded to YouTube for embedding within the Qualtrics webtool. To our knowledge, this is the first time an Internet-based educational video has been used in an NBS-related survey.

#### 2.2.2. Substitution of a Generic Disease X instead of CF

Our experience with previous surveys suggested that community respondents would have varied knowledge about CF, and we grew concerned that this heterogeneity might have an unpredictable influence on summarizing analyses. We therefore substituted for CF a fictitious “Disease X” with symptoms and implications that are very similar to CF. We also felt that the Disease X substitution would be useful for generalizing the study to other genetic conditions included on NBS panels. We developed explanations for autosomal recessive carrier status for Disease X and also created an analog for CRMS/CFSPID called “Mild X”.

#### 2.2.3. Vignettes and Complementary Modes for Preference Questions

We considered a variety of approaches to the vignettes and questions and settled on a method from experimental psychology called an imagination exercise, in which respondents would be presented with a vignette and asked to imagine themselves in the position of a character in the story. The first vignette asked the respondent to imagine that at the same time her/his baby was born, a best friend named Tonya had a baby (Natalie) who was diagnosed with Mild X. Tonya conveys to the respondent all the information about Disease X and Mild X, and then the Nurse Maria character explains about the three policies in Table 1. After the video, the parents were asked to rank the three policies in relation to this scenario. Policies had to be ranked in different positions (first, second, third), but parents were given the option to leave policies unranked.

Next, the respondents were asked “Do you think MOST parents would share your opinion about the policies?” and given two options; “Yes, I think more than half of all parents would share my opinion” and “No, I think that one of the other two policies would be better for most parents *(you will be asked which policy in the next question)*”. Respondents who selected the latter choice were given the policies again and asked “Which policy do you think would be best for most parents of infants with a Mild X result?”

The second vignette reprised the Tonya and Natalie story, but with Natalie diagnosed with genetic carrier status for Disease X, and Nurse Maria explaining carrier status using an animated Punnett square. After the video, respondents were given the same ranking task for placing themselves in Tonya’s position and whether more than half of all parents would share their opinion, and if not then another ranking task for “most parents”. After respondents were asked about their own preferences and their opinions about “most parents”, we used three slider questions to compare how important different values were to each other such as autonomy compared with deferring to a clinician expert.

### 2.3. Sample and Recruitment

Eligible participants were parents of infants up to six months of age regardless of medical history. Fluency in English was required. The study began with a plan to recruit two samples of parents in the state of Wisconsin, beginning with one phase in Milwaukee and then proceeding to another in Madison. The Milwaukee recruiting strategy used fliers at a maternity hospital and clinics that served a poor urban population that is mostly African American. However, due to limited resources for recruiting, the Milwaukee phase served primarily as a pilot testing effort while resulting in six respondents. The Madison phase used recruiting fliers distributed in person at a popular store selling products for infants and at parent education classes located at a hospital with a large and diverse obstetrical population. Participants were told that the survey would take approximately 20 min to complete. As a gratuity for participation, respondents were offered a $10 retail gift card. The contact information for sending the gift card was obtained in a separate survey that was not linked to the subjects’ responses on the survey questions.

### 2.4. Data Management and Statistical Analyses

During the final analyses, descriptive statistics were derived for both parent and child characteristics and frequency information from items evaluating experience with NBS. The proportion of parents ranking each policy first, second, and third was obtained separately for the Mild X and Disease X carrier status scenarios and was reported with Wilson 95% confidence intervals. Descriptive statistics were reported for the continuous value comparison variables and one-way ANOVA was used to evaluate differences in value scores between parents who ranked the Fully Informed, Withhold, and Parents Decide policies first. Statistical significance was determined using two-tailed tests with α = 0.05. All data were analyzed using JMP software (SAS Institute, Cary, NC, USA).

## 3. Results

### 3.1. Sample Characteristics

A total of 213 surveys were started, including 11 in the Milwaukee phase and 202 in the Madison phase. Of those, 35% (4 and 81, respectively) were excluded because the participant stopped early, or generated a response that was too incomplete for analysis, or completed the survey in under 1000 s, suggesting that the subject didn’t watch the entire duration of the video clips. Although these responses contributed some information, we decided as a stringent quality control requirement to accept only complete responses. The final sample included 128 respondents (60.1% of surveys begun). The median duration for the included surveys was 1406 s (IQR = 1007 s), not counting four outliers who left the survey open for more than 30,000 s.

The mother was the respondent in 81.3% of surveys. The median respondent age was 33, while the median infant age was 2 months. Further descriptive data are shown in Table 2. In general, this was a well-educated sample of white married women. However, their knowledge about NBS was limited; 20% of respondents knew nothing or very little about NBS, despite their infant having been screened only a few months before, and 66% wished that they had known more. Thus, information on NBS policy options was new to this group, which we considered an advantage in this survey.

### 3.2. Reaction to Animated Video Survey Format

Reactions to the Nurse Maria videos were favorable among those who finished the survey, with 92% of respondents agreeing or strongly agreeing that they liked the videos, and 98% agreeing or strongly agreeing that the “videos explained things in a way that was easy to understand”. Similarly, 98% agreed or strongly agreed that “the videos were better than reading several long paragraphs”. In view of the well-educated nature of the sample, these responses are a significant finding of this study.

### 3.3. Disclosure Preferences

**Figure 4:**
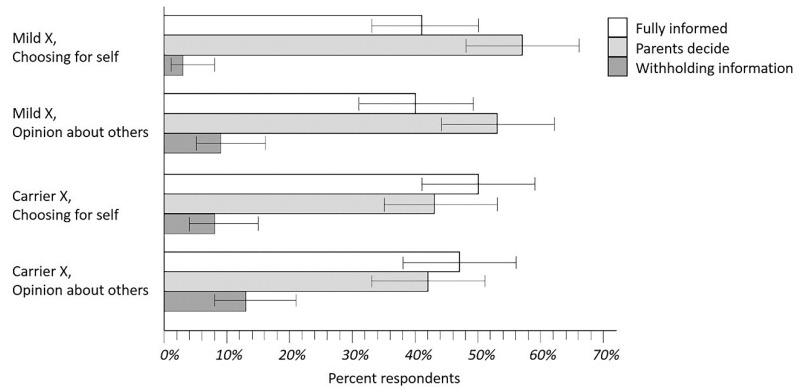
Rankings of disclosure policies for Mild X and Disease X carrier status. Results shown are from respondents asked to rank their preferences for disclosure of policy options that could be implemented by caregivers for either a Mild X condition analogous to CRMS/CFSPID or Disease X like CF. The intent of this exercise was to learn what parents preferred and what they were opposed to as well: clearly, the Withholding Information policy option.

Parents’ rankings of NBS disclosure policies were analyzed separately for both the Mild X and Carrier X scenarios, and for each of two questions: “If you had been in (the vignette), which of the three policies would you have preferred for yourself and your baby?” and “What do you think would be best for most parents of infants with (condition in the vignette)?” The proportion of respondent rankings for these four analyses are shown in Figure 4 where the top-ranked policies are compared (error bars are Wilson confidence intervals). As seen in Figure 4, the Withholding Information policy was obviously less popular than the other two policies. It was more challenging to compare the Fully Informed and Parents Decide policies, but there appeared to be a marginal trend favoring Parent Decide. Several other analyses shed additional light on this situation.

As shown in Figure 4, respondents began by describing what they would have wanted in the Mild X vignette for themselves and their infants. The next three vignettes allowed us to investigate how respondents changed their preferences in different situations. Between 10–25% of parents changed a preference when asked about the Carrier X vignette, or when opining about what would be best for other parents. Five parents (4.2%) who began with a Fully Informed or Parents Decide preference for Mild X answered that Withholding Information would be better for other parents. Nine parents (7.6%) who began with Fully Informed or Parents Decide for Mild X answered that Withholding Information would be better for themselves in the Carrier X vignette.

Respondents’ preferences for the individual policies are compared with the data in Table 2 and other variables obtained through the survey. Respondents who had reported being the primary caregiver for the baby were more likely to vote for the Parents Decide policy (*p* = 0.006, Wilcoxon) or full disclosure policy (*p* = 0.035, Wilcoxon). Respondents with newer infants were less likely to vote in favor of the Parents Decide policy (r = −0.19, *p* < 0.035). A vote in favor of the Withholding Information policy was less likely for parents who recalled being told about the NBS result.

### 3.4. Value Comparison

**Figure 5:**
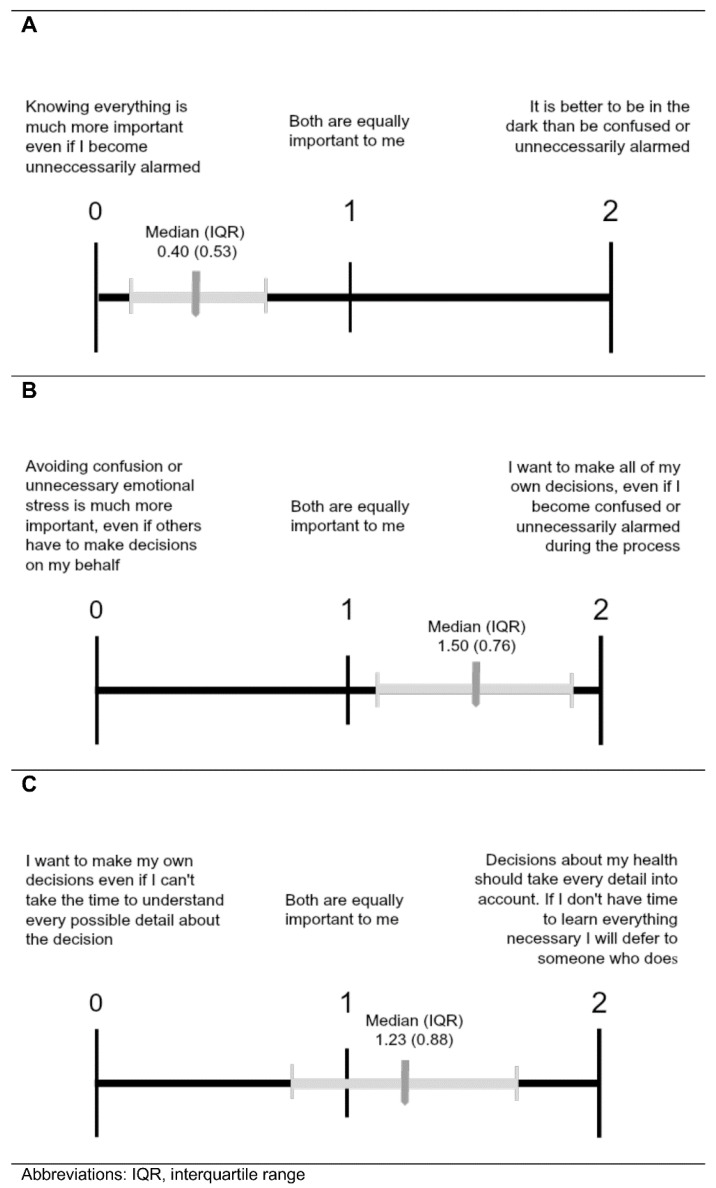
Comparisons of values associated with Fully Informed (**A**), Parents Decide (**B**), and Withholding policies (**C**). Results from parents given value comparison questions to express their views about the importance of the three policy options that may be associated with stress. Values are as described in Figure 3.

Table 3 and Figure 5 depict the three value comparison questions with the latter showing the median (interquartile range) responses for the sample indicated. When comparing the importance of being Fully Informed to reducing emotional distress (Comparison A), parents gave preference to autonomy at the risk of becoming unnecessarily alarmed (Figure 5A). Likewise, when weighing the importance of autonomy in decision-making versus reducing emotional distress (Comparison B), parents preferred the statement consistent with autonomy (Figure 5B). However, when choosing between autonomous decision-making without all pertinent details or allowing someone who is knowledgeable to make decisions (Comparison C), parents showed a slight preference for deferring to someone who knows all necessary information (Figure 5C).

We also explored value scores based on which policy parents ranked first for the Mild X and Disease X carrier status scenarios. All ANOVA results showed significant differences in mean value scores between first-rank policy groups except for Comparison C value scores between first-rank policy groups for Disease X carrier status. For Comparison A (comparing the importance of being fully informed to reducing emotional distress), parents who ranked the Fully Informed policy first most strongly favored being fully informed, followed by those who ranked the Parents Decide policy first, and finally by those who ranked the withhold option first. This pattern was present for first-rank policy groups from both the Mild X and Disease X carrier scenarios. For Mild X, all Hochberg’s GT2 post-hoc tests were significant except the Withholding and Parents Decide groupings for Mild X. Similarly, in Comparison B (comparing the importance of autonomy in decision-making versus reducing emotional distress), parents who gave the Fully Informed policy a first-place ranking most strongly favored autonomy, followed by those who ranked the Parents Decide policy first, and finally by those who ranked the withhold policy first. For Disease X carrier status, all Hochberg’s GT2 post-hoc tests were significant except the Fully Informed and Parents Decide groupings. Compared to those who favored Fully Informed and Parents Decide, parents who ranked Withholding Information first in either scenario had average value scores closest to the withholding statement in Comparisons A and B. Even so, the Withholding Information group averages did not reflect a strong affinity for the withholding statement and tended to indicate a neutral attitude or even slight preference for the opposing statement. Regarding Comparison C (choosing between autonomous decision-making without all pertinent details or allowing someone who is knowledgeable to make decisions), the average value scores for all groups were near the midpoint, with the exception of the Parents Decide groups that slightly favored deferring decision-making to someone else. There were no significant differences in Comparison C value scores for Mild X first-rank policy groups.

## 4. Discussion

There are three main options for policy regarding informing parents about IFs after newborn screening (Table 1), each with its advantages/benefits, disadvantages, and potential value for society. It is ideal for screening policy decisions to incorporate parental perceptions, but the literature provides a mixture of views, along with varying designs and sample populations [15,25,26,27,28,29,30,31,32,33,34]. This study examined preferences for disclosure of NBS results among generally well-educated parents who recently experienced the NBS process, thus seeking their policy preferences in an ideal timeframe when NBS might be fresh in their minds.

In this sample, the policy of withholding IFs (for the purpose of reducing unnecessary distress) was unpopular for both scenarios, although this strategy is often advocated for among clinicians. Applying this policy in NBS as in Norway [4] can be challenged, particularly when there are benefits to knowing your genetic status [23].

In reaction to videos describing a Mild X condition analogous to CRMS/CFSPID, many parents favored policies that kept them fully informed or allowed them to determine whether to receive IFs. This confirms the wisdom of clinical practice recommendations that encourage full disclosure about this condition and the importance of longitudinal follow-up evaluations [11,12,35]. Although incidental findings related to CF were the focus of this study, the generic nature of the video contents might allow these preferences to be informative for disclosure of NBS results beyond CF. If further supported by future study and commentary, the onus would be on NBS programs and their funding providers to mitigate harm following disclosure.

Distinguishing parents’ preferences between the Fully Informed and Parents Decide policies is challenging. In the case of Mild X, more parents ranked the Parents Decide policy option first than ranked the Fully Informed policy first, but for Disease X carrier status, the Fully Informed option was slightly more popular than the Parents Decide policy as a top choice. This may mean that parents believe universal disclosure is less critical for Mild X (CRMS/CFSPID) than for Disease X/CF carrier status, but further study is warranted before such a conclusion could be made. However, the notion that parents may have different preferences for different categories of incidental findings raises the possibility of hybrid policies where certain results are always disclosed, and others are optional.

The value comparison results were largely consistent with policy preferences; parents favored autonomy and being fully informed at the risk of experiencing emotional distress. While we did not probe participants about why they were willing to endure distress, others have reported a sense of obligation or duty among parents in similar situations [31]. Interestingly, even the small number of parents who ranked the Withholding Information policy first did not strongly endorse the withhold statements in value comparison questions. This suggests that perhaps those who favor the withhold policy have high regard for being fully informed and maintaining autonomy but are influenced by other factors to choose the Withholding Information option. Given concerns about the capacity of NBS programs and practitioners to prepare parents to make informed decisions about IFs, we gave special attention to time and resource limitations in value comparison C. Statements in this comparison were written to reflect the possibility that there may not be time to teach parents all relevant information before a health decision needs to be made. On one end, parents could choose to maintain autonomy without all pertinent details, and on the other end, they could defer the health decision to someone who knows all the details. After favoring autonomy in Comparison B, this sample was more inclined to defer decision-making in Comparison C, indicating that knowledge, rather than personal control alone, was important to them. This is a positive indication that parents will understand the difficulties inherent to teaching/learning about IFs as the era of NGS evolves. Although one might argue that parents should not be the sole determinants of the child’s interest in learning about IFs, practical considerations have led to the parent-child dyad as being responsible for this information transfer. In fact, counseling resource limitations make it difficult to engage professional experts in this aspect of NBS follow up communications.

Our study was successful in employing a novel video survey design to deliver complex genetic and clinical information to the public. Thus, it adds to the previous NBS-related research on parental preferences by providing survey methodology that is more user-friendly than reading “several long paragraphs”. Nearly all parents found the contents understandable and more engaging than a conventional written survey format. Although technical expertise is required for video design and creation, this model should be considered for future studies with non-medical populations and perhaps for parent education in association with NBS rather than the traditional brochures. In connection with this, the first video that can be accessed through Figure 1 provides a succinct, 2-min explanation of all aspects of the NBS process.

A limitation of this study is the use of a convenience sample made up mostly of American mothers from a single community that selects for those willing and able to attend a voluntary class in the middle of the day. The sample was disproportionately white, well-educated, and married, all of which limit the generalizability of our results. The homogeneity of the sample was identified during preliminary analyses, after which the research team explored adding more recruitment sites that traditionally serve low-income and minority populations, such as public health departments administering the Special Supplemental Nutrition Program for Women, Infants, and Children (WIC). Unfortunately, we were unable to secure new collaborations. Past research on adults regarding their desire to learn about IFs discovered in genetic testing, including carrier status, has found little association between sociodemographic and literacy factors and preference for disclosure [32,33,34]. Therefore, our results may be similar with a more diverse population, although this remains a topic for further study.

Despite these limitations in generalizability, our study extends previous observations about parental preferences [15,26,27,28,29,30] by its comparison of reactions to information disclosure about a potentially severe disease such as CF (Disease X) with a mild condition such as CRMS/CFSPID, and by incorporating three policy options into the survey, in addition to contributing a user-friendly video survey option to the range of methodologies available. Although there was less interest in the Parents Decide disclosure option with Disease X, the respondents clearly were opposed to withholding information on carrier status, even if the condition is mild. Policymakers need to keep this in mind as NGS-based screening expands, requiring both ethical [18,23] and practical issues [17] need to be addressed. Thus, another implication of our study is that valuable parental input can be obtained about policy options with user-friendly, efficient methods prior to widespread implementation of NGS. Although some may argue that parental input should not be considered in formulating disclosure policies about IFs from screening tests, people participating in healthcare systems have a right to be engaged in the sharing of health-related, relevant knowledge, and NBS is a hybrid of public health and healthcare. The strong preference for autonomy that was identified in this survey underscores the importance of that ethical principle.

## Figures and Tables

**Table 1:** Disclosure Policy Definitions and Associated Advantages, Disadvantages, and Added Values.

Policy	Definition	Advantages (Benefits)	Disadvantages	Added Value ^a^
Fully Informed	Incidental findings always disclosed to parents	No information is withheld from parents	Requires more time that could be devoted to other health care needs Potential for confusion, misconceptions, emotional stress	Being fully informed
Withholding Information	Incidental findings with very minimal or no risk to health are withheld from parents	Reduces parental confusion and risk of emotional problems	Parents not consulted about information that is part of them and their family	Reducing emotional stress
Parents Decide	Parents are counseled just prior to results communication, and then make a decision about how much information to receive about incidental genetic findings	Parents are in control of the screening results that affect their baby and can determine if they want full details if they are willing to risk emotional distress	Requires more time that could be devoted to other health care needs Curiosity may lead to the parents asking questions about details they don’t actually want or need to know	Autonomy is preserved (freedom of choice) ^b^

^a^ Values associated with policies were not directly presented to participants. ^b^ Preserving the principle of autonomy is ethically sound.

**Table 2:** Sample Characteristics for 128 Respondents.

Characteristic	N	(%)
Married or stable relationship	123	(96.1)
First-time parent	93	(72.7)
Infant born >2 weeks before due date	19	(14.8)
NICU stay >2 days	11	(8.6)
Race and/or ethnicity of respondent		
White	112	(87.5)
Non-White	11	(8.6)
Missing response	5	(3.9)
Education		
High school	11	(9)
Undergraduate degree	62	(48.4)
Postgraduate degree	52	(40.6)
Missing response	3	(2.3)
Health literacy (need help reading…)		
never	86	(67.2)
rarely	28	(21.9)
sometimes	8	(6.3)
often	1	(0.8)
always	1	(0.8)
not answered	4	(3.1)

**Table 3:** Value Score Results for First-rank Policy Groups.

	First-Rank Policy Group			*p*-Value *
	Fully Informed	Withholding Information	Parents Decide	
Comparison A (comparing the importance of being fully informed to reducing distress)				
Mild X scenario				
mean (std. dev.)	0.28 (0.33)	0.76 (0.78)	0.58 (0.44)	<0.001 *
median (IRQ)	0.17 (0.42)	0.73 (1.55)	0.48 (0.52)	
Disease X carrier status scenario				
mean (std. dev.)	0.32 (0.38)	0.96 (0.61)	0.55 (0.38)	<0.001 *
median (IRQ)	0.19 (0.40)	1.0 (0.92)	0.47 (0.44)	
Comparison B (comparing the importance of autonomy in decision-making versus reducing emotional distress)				
Mild X scenario				
mean (std. dev.)	1.61 (0.50)	0.76 (0.74)	1.37 (0.46)	<0.001 *
median (IRQ)	1.76 (0.63)	0.58 (1.10)	1.40 (0.61)	
Disease X carrier status scenario				
mean (std. dev.)	1.51 (0.56)	1.03 (0.40)	1.45 (0.43)	0.015 *
median (IRQ)	1.69 (0.71)	1.05 (0.66)	1.46 (0.58)	
Comparison C (comparing autonomous Decision-making without all details to allowing experts to make decisions)				
Mild X scenario				
mean (std. dev.)	0.97 (0.62)	0.95 (0.83)	1.27 (0.51)	0.008 *
median (IRQ)	1.01 (1.00)	1.01 (1.60)	1.33 (0.61)	
Disease X carrier status scenario				
mean (std. dev.)	1.04 (0.61)	1.08 (0.61)	1.25 (0.54)	0.092
median (IRQ)	1.13 (1.01)	1.23 (0.92)	1.33 (0.65)	

* One-way ANOVA.

## Abbreviations

cystic fibrosis	CF
CFTR-related metabolic syndrome	CRMS
cystic fibrosis screen positive inconclusive diagnosis	CFSPID
newborn screening	NBS
next generation sequencing	NGS
incidental findings	IFs
confidence interval	CI

## References

[1] Grosse S.D., Boyle C.A., Botkin J.R., Comeau A.M., Kharrazi M., Rosenfeld M., Wilfond B.S., CDC (2004). Newborn screening for cystic fibrosis: Evaluation of benefits and risks and recommendations for state newborn screening programs. Morb. Mortal. Wkly. Rep. Recomm. Rep..

[2] Pederzini F., Armani P., Barbato A., Borgo G., Castellani E., Girella M., Manfrini G., Olivieri D., Righetti G., Zamboni C. (1983). Newborn screening for cystic fibrosis. The methods compared on 229,626 newborns tested in 8 years in the Veneto Region. Riv. Ital. Pediatr.-Ital. J. Pediatrics.

[3] Scotet V., Gutierrez H., Farrell P.M. (2020). Newborn screening for CF across the globe—Where is it worthwhile?. Int. J. Neonatal Screen.

[4] Lundman E., Gaup H.J., Bakkeheim E., Olafsdottir E.J., Rootwelt T., Storrøsten O.T., Pettersen R.D. (2016). Implementation of newborn screening for cystic fibrosis in Norway. Results from the first three years. J. Cyst. Fibros..

[5] Hammond K.B., Abman S.H., Sokol R.J., Accurso F.J. (1991). Efficacy of statewide neonatal screening for cystic fibrosis by assay of trypsinogen concentrations. N. Engl. J. Med..

[6] Gregg R.G., Wilfond B.S., Farrell P.M., Laxova A., Hassemer D., Mischler E.H. (1993). Application of DNA analysis in a population-screening program for neonatal diagnosis of cystic fibrosis (CF): Comparison of screening protocols. Am. J. Hum. Genet..

[7] Comeau A.M., Parad R.B., Dorkin H.L., Dovey M., Gerstle R., Haver K., Lapey A., O’Sullivan B.P., Waltz D.A., Zwerdling R.G. (2004). Population-based newborn screening for genetic disorders when multiple mutation DNA testing is incorporated: A cystic fibrosis newborn screening model demonstrating increased sensitivity but more carrier detections. Pediatrics.

[8] Sicko R.J., Stevens C.F., Hughes E.E., Leisner M., Ling H., Saavedra-Matiz C.A., Caggana M., Kay D.M. (2021). Validation of a custom next-generation sequencing assay for cystic fibrosis newborn screening. Int. J. Neonatal Screen.

[9] Baker M.W., Atkins A.E., Cordovado S.K., Hendrix M., Earley M.C., Farrell P.M. (2016). Improving newborn screening for cystic fibrosis using next-generation sequencing technology: A technical feasibility study. Genet. Med..

[10] Hughes E.E., Stevens C.F., Saavedra-Matiz C.A., Tavakoli N.P., Krein L.M., Parker A., Zhang Z., Maloney B., Vogel B., DeCelie-Germana J. (2016). Clinical sensitivity of cystic fibrosis mutation panels in a diverse population. Hum. Mutat..

[11] Ren C.L., Borowitz D.S., Gonska T., Howenstine M.S., Levy H., Massie J., Milla C., Munck A., Southern K.W. (2017). Cystic fibrosis transmembrane conductance regulator-related metabolic Syndrome and cystic fibrosis screen positive, inconclusive diagnosis. J. Pediatrics.

[12] Munck A., Mayell S.J., Winters V., Shawcross A., Derichs N., Parad R., Barben J., Southern K.W., ECFS Neonatal Screening Working Group (2015). cystic fibrosis screen positive, inconclusive diagnosis (CFSPID): A new designation and management recommendations for infants with an inconclusive diagnosis following newborn screening. J. Cyst. Fibros..

[13] Berg J.S., Agrawal P.B., Bailey DBJr Beggs A.H., Brenner S.E., Brower A.M., Cakici J.A., Ceyhan-Birsoy O., Chan K., Chen F., Currier R.J. (2017). Newborn Sequencing in Genomic Medicine and Public Health. Pediatrics.

[14] Howard H.C., Knoppers B.M., Cornel M.C., Wright Clayton E., Sénécal K., Borry P. (2015). Whole-genome sequencing in newborn screening? A statement on the continued importance of targeted approaches in newborn screening programmes. Eur. J. Hum. Genet..

[15] Ulph F., Cullinan T., Qureshi N., Kai J. (2015). Parents’ responses to receiving sickle cell or cystic fibrosis carrier results for their child following newborn screening. Eur. J. Hum. Genet..

[16] Castellani C., Duff A.J.A., Bell S.C., Heijerman H.G.M., Munck A., Ratjen F., Sermet-Gaudelus I., Southern K.W., Barben J., Flume P.A. (2018). ECFS best practice guidelines: The 2018 revision. J. Cyst. Fibros..

[17] CLSI (2019). Newborn Screening for Cystic Fibrosis.

[18] Goldenberg A.J., Sharp R.R. (2012). The ethical hazards and programmatic challenges of genomic newborn screening. JAMA.

[19] Farrell M., Certain L., Farrell P. (2001). Genetic counseling and risk communication services of newborn screening programs. Arch. Pediatrics Adolesc. Med..

[20] Farrell M.H., Christopher S.A. (2013). Frequency of high-quality communication behaviors used by primary care providers of heterozygous infants after newborn screening. Patient Educ. Couns..

[21] La Pean A., Farrell M.H., Eskra K.L., Farrell P.M. (2013). Effects of immediate telephone follow-up with providers on sweat chloride test timing after cystic fibrosis newborn screening identifies a single mutation. J. Pediatrics.

[22] Christopher S.A., Collins J.L., Farrell M.H. (2012). Effort required to contact primary care providers after newborn screening identifies sickle cell trait. J. Natl. Med. Assoc..

[23] Farrell P.M., Langfelder-Schwind E., Farrell M.H. (2021). Challenging the dogma of the healthy heterozygote: Implications for newborn screening policies and practices. Mol. Genet. Metab..

[24] Ciske D.J., Haavisto A., Laxova A., Rock L.Z.M., Farrell P.M. (2001). Genetic counseling and neonatal screening for cystic fibrosis: An assessment of the communication process. Pediatrics.

[25] La Pean A., Collins J.L., Christopher S.A., Eskra K.L., Roedl S.J., Tluczek A., Farrell M.H. (2012). A qualitative secondary evaluation of statewide follow-up interviews for abnormal newborn screening results for cystic fibrosis and sickle cell hemoglobinopathy. Genet. Med..

[26] Collins J.L., La Pean A., O’Tool F., Eskra K.L., Roedl S.J., Tluczek A., Farrell M.H. (2013). Factors that influence parents’ experiences with results disclosure after newborn screening identifies genetic carrier status for cystic fibrosis or sickle cell hemoglobinopathy. Patient Educ. Couns..

[27] Lang C.W., McColley S.A., Lester L.A., Ross L.F. (2011). Parental understanding of newborn screening for cystic fibrosis after a negative sweat-test. Pediatrics.

[28] Fernandez C.V., Bouffet E., Malkin D., Jabado N., O’Connell C., Avard D., Knoppers B.M., Ferguson M., Boycott K.M., Sorensen P.H. (2014). Attitudes of parents toward the return of targeted and incidental genomic research findings in children. Genet. Med..

[29] Harris E.D., Ziniel S.I., Amatruda J.G., Clinton C.M., Savage S.K., Taylor P.L., Huntington N.L., Green R.C., Holm I.A. (2012). The beliefs, motivations, and expectations of parents who have enrolled their children in a genetic biorepository. Genet. Med..

[30] Ziniel S., Savage S.K., Huntington N., Amatruda J., Green R.C., Weitzman E.R., Taylor P., Holm I.A. (2014). Parents’ preferences for return of results in pediatric genomic research. Public Health Genom..

[31] Christensen K.D., Savage S.K., Huntington N.L., Weitzman E.R., Ziniel S.I., Bacon P.L., Cacioppo C.N., Green R.C., Holm I.A. (2017). Preferences for the return of individual results from research on pediatric biobank samples. J. Empir. Res. Hum. Res. Ethics.

[32] Anderson J.A., Meyn M.S., Shuman C., Shaul R.Z., Mantella L.E., Szego M.J., Bowdin S., Monfared N., Hayeems R.Z. (2017). Parents perspectives on whole genome sequencing for their children: Qualified enthusiasm?. J. Med. Ethics.

[33] Rini C., Khan C.M., Moore E., Roche M.I., Evans J.P., Berg J.S., Powell B.C., Corbie-Smith G., Foreman A.K.M., Griesemer I. (2018). The who, what and why of research participants’ intentions to request a broad range of secondary findings in a diagnostic genomic sequencing study. Genet. Med..

[34] Haga S.B., O’Daniel J.M., Tindall G.M., Lipkus I.R., Agans R. (2011). Public attitudes towards ancillary information revealed by pharmacogenetic testing under limited information conditions. Genet. Med..

[35] Barben J., Castellani C., Munck A., Davies J.C., de Winter-de Groot K.M., Gartner S., Kashirskaya N., Linnane B., Mayell S.J., McColley S. (2021). Updated guidance on the management of children with cystic fibrosis transmembrane conductance regulator-related metabolic syndrome/cystic fibrosis screen positive, inconclusive diagnosis (CRMS/CFSPID). J. Cyst. Fibros..

